# Tetra­kis(μ_2_-2-methyl-3,5-dinitro­benzoato-κ^2^ *O* ^1^:*O* ^1′^)bis­[aqua­copper(II)] tetra­hydrate

**DOI:** 10.1107/S1600536811000547

**Published:** 2011-01-15

**Authors:** Muhammad Danish, Sabiha Ghafoor, M. Nawaz Tahir, Nazir Ahmad, Mehwish Nisa

**Affiliations:** a Department of Chemistry, University of Sargodha, Sargodha, Pakistan; b Department of Physics, University of Sargodha, Sargodha, Pakistan

## Abstract

The title compound, [Cu_2_(C_8_H_5_N_2_O_6_)_4_(H_2_O)_2_]·4H_2_O, forms a centrosymmetric paddle-wheel-type dimer with an intra­molecular Cu⋯Cu distance of 2.6540 (4) Å. The Cu^II^ atom is in a square-pyramidal coordination environment formed by four O atoms of four carboxyl­ate groups and one water mol­ecule, which is located in the apical position. The carboxyl­ate groups are twisted relative to the benzene rings by 11.09 (16) and 45.55 (19)°. The nitro groups are not coplanar with the parent aromatic rings [dihedral angles = 16.2 (3)–51.45 (14)°]. O—H⋯O hydrogen bonds between the coordinated water mol­ecules and one of the nitro groups, as well as π–π stacking inter­actions [centroid–centroid distance = 3.5764 (12) Å] between the benzene rings, assemble the complex mol­ecules into a one-dimensional polymeric structure which is further extended into a three-dimensional polymeric network *via* O—H⋯O hydrogen bonds involving the water molecules of crystallization.

## Related literature

For related crystal structures, see: Chen *et al.* (2007 ▶); Danish *et al.* (2010 ▶); Moncol *et al.* (2006 ▶); Stachova *et al.* (2004 ▶); Viossat *et al.* (2005 ▶).
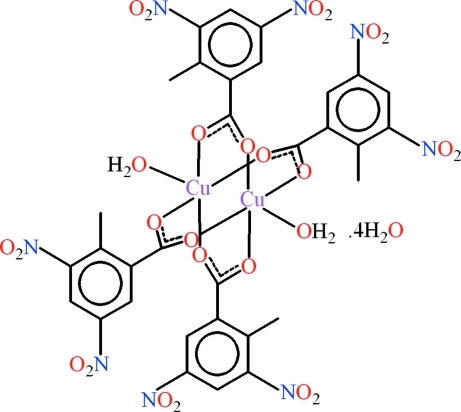


## Experimental

### 

#### Crystal data

[Cu_2_(C_8_H_5_N_2_O_6_)_4_(H_2_O)_2_]·4H_2_O
*M* *_r_* = 1135.76
Monoclinic, 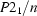

*a* = 8.9757 (3) Å
*b* = 22.5582 (8) Å
*c* = 11.2698 (3) Å
β = 104.443 (1)°
*V* = 2209.75 (12) Å^3^
*Z* = 2
Mo *K*α radiation
μ = 1.08 mm^−1^
*T* = 296 K
0.30 × 0.24 × 0.22 mm

#### Data collection

Bruker Kappa APEXII CCD diffractometer
Absorption correction: multi-scan (*SADABS*; Bruker, 2005 ▶) *T*_min_ = 0.743, *T*_max_ = 0.782
21133 measured reflections
5449 independent reflections
4344 reflections with *I* > 2σ(*I*)
*R*_int_ = 0.035

#### Refinement

*R*[*F* ^2^ > 2σ(*F* ^2^)] = 0.034
*wR*(*F* ^2^) = 0.088
*S* = 1.02
5449 reflections
345 parameters
6 restraints
H atoms treated by a mixture of independent and constrained refinement
Δρ_max_ = 0.33 e Å^−3^
Δρ_min_ = −0.28 e Å^−3^

### 

Data collection: *APEX2* (Bruker, 2009 ▶); cell refinement: *SAINT* (Bruker, 2009 ▶); data reduction: *SAINT*; program(s) used to solve structure: *SHELXS97* (Sheldrick, 2008 ▶); program(s) used to refine structure: *SHELXL97* (Sheldrick, 2008 ▶); molecular graphics: *ORTEP-3 for Windows* (Farrugia, 1997 ▶) and *PLATON* (Spek, 2009 ▶); software used to prepare material for publication: *WinGX* (Farrugia, 1999 ▶) and *PLATON*.

## Supplementary Material

Crystal structure: contains datablocks global, I. DOI: 10.1107/S1600536811000547/gk2339sup1.cif

Structure factors: contains datablocks I. DOI: 10.1107/S1600536811000547/gk2339Isup2.hkl

Additional supplementary materials:  crystallographic information; 3D view; checkCIF report

## Figures and Tables

**Table 1:** Selected bond lengths (Å)

Cu1—O1	1.9616 (16)
Cu1—O7	1.9749 (16)
Cu1—O8	1.9637 (15)
Cu1—O13	2.0914 (19)
Cu1—O2^i^	1.9595 (16)

Symmetry code: (i) 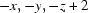.

**Table 2:** Hydrogen-bond geometry (Å, °)

*D*—H⋯*A*	*D*—H	H⋯*A*	*D*⋯*A*	*D*—H⋯*A*
O13—H13*A*⋯O14^ii^	0.80 (3)	1.84 (3)	2.641 (3)	175 (3)
O13—H13*B*⋯O10^iii^	0.77 (2)	2.22 (2)	2.838 (2)	138 (3)
O14—H14*A*⋯O15^iv^	0.81 (3)	1.95 (3)	2.758 (4)	172 (3)
O14—H14*B*⋯O7	0.80 (3)	2.51 (4)	3.182 (3)	143 (4)
O15—H15*A*⋯O12^v^	0.83 (4)	2.16 (4)	2.953 (4)	161 (4)
O15—H15*B*⋯O5^vi^	0.84 (3)	2.17 (2)	2.996 (4)	168 (4)
O15—H15*B*⋯O6^vi^	0.84 (3)	2.60 (4)	3.273 (4)	138 (4)

Symmetry codes: (ii) 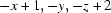; (iii) 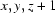; (iv) 
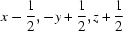
; (v) 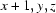; (vi) 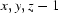.

**Fig. 1.**
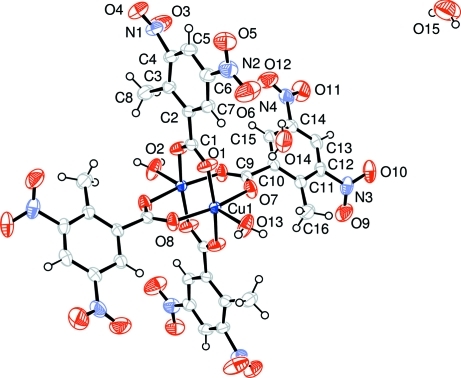
View of the title compound with the atom numbering scheme. The displacement ellipsoids are drawn at the 50% probability level. H-atoms are shown as small spheres of arbitrary radii.

**Fig. 2.**
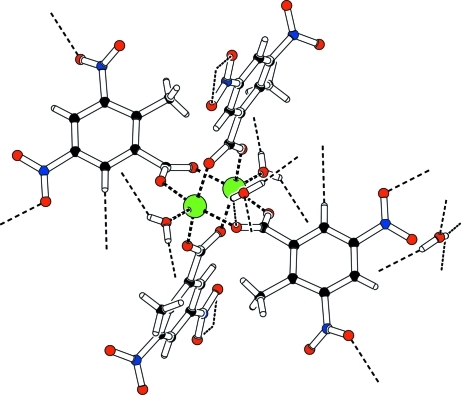
The partial packing (PLATON; Spek, 2009) which shows the H-bonding mode with neighbouring molecules through dotted lines except which are present in coordination sphere.

**Fig. 3.**
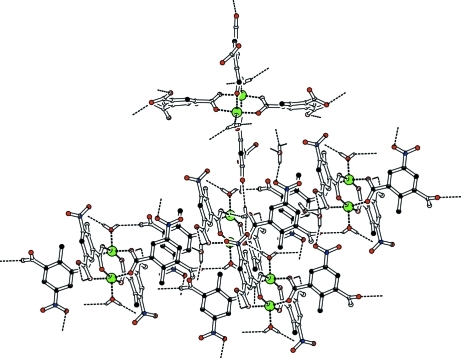
The partial packing (PLATON; Spek, 2009) which shows that molecules form three-dimensional polymeric network due to strong intermolecular H-bondings.

## supplementary crystallographic information

### Comment

Recently we have reported the synthesis and crystal structure of tetrakis(µ-2-methylbenzoato-κ^2^*O*:*O*^'^)bis[(methanol-O)copper(II)]. In continuation to our interest with the metal carboxylate chemistry, the title compound (I), (Fig. 1) is being reported here.

The crystal structure of (II) *i.e.*, diaqua-tetrakis(µ_2_-2,3,5-tri-iodobenzoato-k^2^O:*O*^'^)-di-copper(ii) bis(methanol-κ*O*)-tetrakis(µ_2_-2,3,5-tri-iodobenzoato- κ_2_O:*O*^'^)-di-copper(ii) methanol solvate (Chen *et al.*, 2007), (III) *i.e.*, tetrakis(µ_2_-2-nitrobenzoato-*O*,*O*^'^)-bis(aqua-copper(ii)) ethanol solvate (Moncol *et al.*, 2006), (IV) *i.e.*, tetrakis(µ_2_-2-(3-trifluoromethylphenyl)aminonicotinato)-diaqua-di-copper(ii) *N*,*N*-dimethylformamide solvate (Viossat *et al.*, 2005) and (V) *i.e.*, diaqua-tetrakis(2-nitrobenzoato-O,*O*^'^)-di-copper(ii) dihydrate (Stachova *et al.*, 2004) have been published which are related to the title compound (I) due to the coordination around copper.

The structure of (I) is centrosymmetric with a square pyramidal coordination around the Cu-atoms. Four oxygen atoms (O1/O2^i^/O7/O8; i = -*x*, -*y*, -*z* + 2) from four carboxylate groups are in plane [r. m.s deviation of 0.0014 Å] and coordinated to copper(II) with bond distance of 1.9593 (15) – 1.9749 (15) Å. The Cu—O [2.0909 (17) Å] bond of water molecule is longer as compared to Cu—O bonds of carboxylate groups. The Cu atom is at a distance of 0.2189 (8) Å from the mean square plane of carboxylate O-atoms. The separation of Cu—Cu is 2.6540 (4) Å. These values are in agreement with the reported structures [Table 1]. The toluene groups A (C2—C8) and B (C10—C16) are almost planar with r. m.s deviation of 0.0524 Å and 0.0363 Å and are nearly perpendicular to each other. The dihedral angle between A/B is 89.33 (6)°. The carboxylate moiety C (O1/C1/O2), nitro groups D (O3/N1/O4) and E (O5/N2/O6) are oriented at dihedral angles of 11.09 (16), 51.45 (14) and 45.30 (33)°, respectively with the parent toluene (A) moiety. In the other ligand, the carboxylate moiety F (O7/C9/O8^i^), nitro groups G (O9/N3/O10) and H (O11/N4/O12) are oriented at dihedral angles of 45.55 (19), 40.43 (23) and 16.20 (34)°, respectively with the parent toluene (B) moiety. The molecules are stabilized in the form of three-dimensional polymeric network due to strong H-bonding (Table 2, Fig. 2, Fig. 3). There also exists a π···π interaction between the benzene rings (C10—C15) with centroid-to- centroid distance of 3.5764 (12) Å.

### Experimental

Aqueous solutions of sodium salt of 3,5-dinitro- *o*-toluic acid (0.496 g, 2.0 mol) and copper chloride dihydrate (0.170 g, 1.0 mmol) were prepared separately. Both solutions were mixed in 100 ml round-bottom flask at room temperature. On mixing dirty green precipitate was formed at the begining and it disappeared on continuous stirring of the reaction mixture for 4 h. The reaction mixture was filtered and the filtrate was concentrated by heating for few minutes. The concentrated solution was kept at room temperature for 72 h to afford greenish blue prisms of of the title compound.

### Refinement

The coordinates of H-atoms of water molecules were refined with the distance restraints imposed on the O-H distances and with *U*_iso_(H) = 1.2 *U*_eq_(O),. The C-bound H atoms were positioned geometrically (C–H = 0.93–0.96 Å) and refined as riding with *U*_iso_(H) = *xU*_eq_(C), where *x* = 1.5 for methyl and *x* = 1.2 for other H-atoms.

### Crystal data

**Table:**

[Cu_2_(C_8_H_5_N_2_O_6_)_4_(H_2_O)_2_]·4H_2_O	*F*(000) = 1156
*M**_r_* = 1135.76	*D*_x_ = 1.707 Mg m^−^^3^
Monoclinic, *P*2_1_/*n*	Mo *K*α radiation, λ = 0.71073 Å
Hall symbol: -P 2yn	Cell parameters from 4344 reflections
*a* = 8.9757 (3) Å	θ = 2.1–28.3°
*b* = 22.5582 (8) Å	µ = 1.08 mm^−^^1^
*c* = 11.2698 (3) Å	*T* = 296 K
β = 104.443 (1)°	Prism, blue
*V* = 2209.75 (12) Å^3^	0.30 × 0.24 × 0.22 mm
*Z* = 2	

### Data collection

**Table:**

Bruker Kappa APEXII CCD diffractometer	5449 independent reflections
Radiation source: fine-focus sealed tube	4344 reflections with *I* > 2σ(*I*)
graphite	*R*_int_ = 0.035
Detector resolution: 7.50 pixels mm^-1^	θ_max_ = 28.3°, θ_min_ = 2.1°
ω scans	*h* = −11→11
Absorption correction: multi-scan (*SADABS*; Bruker, 2005)	*k* = −30→30
*T*_min_ = 0.743, *T*_max_ = 0.782	*l* = −15→9
21133 measured reflections	

### Refinement

**Table:**

Refinement on *F*^2^	Primary atom site location: structure-invariant direct methods
Least-squares matrix: full	Secondary atom site location: difference Fourier map
*R*[*F*^2^ > 2σ(*F*^2^)] = 0.034	Hydrogen site location: inferred from neighbouring sites
*wR*(*F*^2^) = 0.088	H atoms treated by a mixture of independent and constrained refinement
*S* = 1.02	*w* = 1/[σ^2^(*F*_o_^2^) + (0.0398*P*)^2^ + 0.8547*P*] where *P* = (*F*_o_^2^ + 2*F*_c_^2^)/3
5449 reflections	(Δ/σ)_max_ = 0.001
345 parameters	Δρ_max_ = 0.33 e Å^−^^3^
6 restraints	Δρ_min_ = −0.28 e Å^−^^3^

### Special details

**Table:**

Geometry. Bond distances, angles *etc*. have been calculated using the rounded fractional coordinates. All su's are estimated from the variances of the (full) variance-covariance matrix. The cell e.s.d.'s are taken into account in the estimation of distances, angles and torsion angles
Refinement. Refinement of *F*^2^ against ALL reflections. The weighted *R*-factor *wR* and goodness of fit *S* are based on *F*^2^, conventional *R*-factors *R* are based on *F*, with *F* set to zero for negative *F*^2^. The threshold expression of *F*^2^ > σ(*F*^2^) is used only for calculating *R*-factors(gt) *etc*. and is not relevant to the choice of reflections for refinement. *R*-factors based on *F*^2^ are statistically about twice as large as those based on *F*, and *R*- factors based on ALL data will be even larger.

### Fractional atomic coordinates and isotropic or equivalent isotropic displacement parameters (Å^2^)

**Table:**

	*x*	*y*	*z*	*U*_iso_*/*U*_eq_	
Cu1	0.14133 (3)	−0.01627 (1)	1.05844 (2)	0.0264 (1)	
O1	0.17167 (19)	0.06719 (7)	1.10736 (17)	0.0528 (6)	
O2	−0.06417 (19)	0.09368 (7)	1.00866 (15)	0.0474 (5)	
O3	−0.1238 (3)	0.32675 (9)	0.9849 (2)	0.0826 (9)	
O4	−0.0417 (3)	0.36016 (8)	1.1678 (2)	0.0701 (8)	
O5	0.5065 (2)	0.29719 (9)	1.30010 (18)	0.0624 (7)	
O6	0.5609 (2)	0.20680 (10)	1.2665 (3)	0.0901 (9)	
O7	0.19256 (18)	0.00393 (8)	0.90275 (14)	0.0461 (5)	
O8	0.04616 (18)	−0.02830 (8)	1.19572 (13)	0.0445 (5)	
O9	0.2910 (2)	−0.08610 (9)	0.45982 (19)	0.0680 (8)	
O10	0.3870 (2)	−0.01075 (10)	0.38692 (16)	0.0623 (7)	
O11	0.0204 (3)	0.15433 (11)	0.34615 (17)	0.0807 (9)	
O12	−0.0072 (3)	0.18886 (9)	0.51609 (19)	0.0792 (9)	
O13	0.3664 (2)	−0.04335 (10)	1.14010 (15)	0.0592 (7)	
N1	−0.0457 (3)	0.32313 (9)	1.0898 (2)	0.0496 (7)	
N2	0.4706 (2)	0.24745 (10)	1.2591 (2)	0.0518 (7)	
N3	0.3119 (2)	−0.03328 (11)	0.45226 (17)	0.0472 (7)	
N4	0.0329 (3)	0.15041 (10)	0.45558 (18)	0.0510 (7)	
C1	0.0668 (2)	0.10469 (9)	1.07283 (18)	0.0321 (6)	
C2	0.1073 (2)	0.16812 (8)	1.10974 (17)	0.0309 (6)	
C3	−0.0028 (3)	0.21412 (9)	1.08940 (18)	0.0347 (6)	
C4	0.0571 (3)	0.27102 (9)	1.1212 (2)	0.0373 (6)	
C5	0.2074 (3)	0.28347 (10)	1.1774 (2)	0.0400 (7)	
C6	0.3078 (3)	0.23623 (10)	1.19871 (19)	0.0377 (7)	
C7	0.2606 (3)	0.17952 (9)	1.16425 (18)	0.0350 (6)	
C8	−0.1731 (3)	0.20411 (11)	1.0466 (3)	0.0523 (8)	
C9	0.0935 (2)	0.02030 (8)	0.81066 (18)	0.0308 (6)	
C10	0.1378 (2)	0.03256 (9)	0.69218 (17)	0.0303 (6)	
C11	0.2268 (2)	−0.00702 (9)	0.64135 (18)	0.0333 (6)	
C12	0.2367 (2)	0.00750 (10)	0.52214 (19)	0.0353 (6)	
C13	0.1764 (2)	0.05764 (10)	0.45945 (18)	0.0379 (7)	
C14	0.0982 (2)	0.09566 (10)	0.51708 (18)	0.0355 (6)	
C15	0.0760 (2)	0.08360 (9)	0.63150 (18)	0.0341 (6)	
C16	0.3062 (3)	−0.05950 (12)	0.7111 (2)	0.0517 (8)	
O14	0.4622 (3)	0.09823 (11)	0.9851 (3)	0.0827 (10)	
O15	0.8508 (3)	0.29497 (14)	0.3805 (4)	0.1215 (14)	
H5	0.24001	0.32188	1.20013	0.0480*	
H7	0.33171	0.14872	1.17755	0.0421*	
H8A	−0.20137	0.19949	0.95920	0.0784*	
H8B	−0.22654	0.23750	1.06903	0.0784*	
H8C	−0.20028	0.16893	1.08435	0.0784*	
H13	0.18803	0.06549	0.38126	0.0455*	
H13A	0.419 (3)	−0.0616 (13)	1.105 (2)	0.0711*	
H13B	0.389 (4)	−0.0520 (14)	1.2082 (17)	0.0711*	
H15	0.01980	0.10960	0.66751	0.0409*	
H16A	0.34251	−0.04933	0.79617	0.0776*	
H16B	0.39179	−0.07080	0.67924	0.0776*	
H16C	0.23523	−0.09198	0.70265	0.0776*	
H14A	0.428 (5)	0.1281 (12)	0.948 (3)	0.0993*	
H14B	0.410 (4)	0.0722 (14)	1.000 (4)	0.0993*	
H15A	0.910 (5)	0.2681 (17)	0.414 (4)	0.1457*	
H15B	0.755 (2)	0.291 (2)	0.352 (4)	0.1457*	

### Atomic displacement parameters (Å^2^)

**Table:**

	*U*^11^	*U*^22^	*U*^33^	*U*^12^	*U*^13^	*U*^23^
Cu1	0.0288 (1)	0.0251 (1)	0.0263 (1)	0.0023 (1)	0.0088 (1)	0.0015 (1)
O1	0.0498 (9)	0.0261 (8)	0.0741 (12)	0.0047 (7)	−0.0001 (8)	−0.0090 (8)
O2	0.0451 (9)	0.0282 (8)	0.0629 (10)	0.0005 (7)	0.0023 (8)	−0.0085 (7)
O3	0.0964 (17)	0.0585 (13)	0.0761 (14)	0.0322 (12)	−0.0102 (12)	0.0002 (11)
O4	0.0857 (15)	0.0430 (11)	0.0871 (14)	0.0112 (10)	0.0318 (12)	−0.0168 (10)
O5	0.0623 (12)	0.0517 (11)	0.0660 (12)	−0.0168 (9)	0.0024 (9)	−0.0118 (9)
O6	0.0472 (12)	0.0617 (14)	0.147 (2)	0.0052 (10)	−0.0027 (13)	−0.0153 (14)
O7	0.0371 (8)	0.0709 (11)	0.0326 (8)	0.0032 (8)	0.0129 (7)	0.0138 (8)
O8	0.0399 (9)	0.0664 (11)	0.0310 (8)	0.0109 (8)	0.0160 (6)	0.0103 (7)
O9	0.0780 (14)	0.0575 (13)	0.0740 (13)	0.0053 (11)	0.0294 (11)	−0.0234 (10)
O10	0.0497 (10)	0.1042 (16)	0.0394 (9)	−0.0029 (10)	0.0233 (8)	−0.0130 (10)
O11	0.1012 (17)	0.1012 (18)	0.0450 (10)	0.0273 (14)	0.0284 (11)	0.0341 (11)
O12	0.126 (2)	0.0554 (12)	0.0644 (13)	0.0282 (13)	0.0394 (13)	0.0195 (10)
O13	0.0469 (10)	0.0925 (15)	0.0348 (9)	0.0330 (10)	0.0037 (8)	−0.0047 (10)
N1	0.0556 (13)	0.0312 (10)	0.0638 (14)	0.0042 (9)	0.0181 (11)	−0.0027 (10)
N2	0.0475 (12)	0.0485 (12)	0.0554 (13)	−0.0071 (10)	0.0054 (10)	−0.0010 (10)
N3	0.0392 (10)	0.0671 (15)	0.0365 (10)	0.0007 (10)	0.0116 (8)	−0.0167 (10)
N4	0.0581 (13)	0.0568 (13)	0.0418 (11)	0.0040 (10)	0.0192 (10)	0.0180 (10)
C1	0.0438 (12)	0.0261 (10)	0.0300 (10)	−0.0011 (8)	0.0162 (9)	−0.0007 (8)
C2	0.0423 (11)	0.0244 (9)	0.0288 (9)	−0.0011 (8)	0.0144 (8)	−0.0003 (8)
C3	0.0439 (12)	0.0298 (10)	0.0324 (10)	0.0012 (9)	0.0134 (9)	−0.0002 (8)
C4	0.0473 (12)	0.0274 (10)	0.0387 (11)	0.0048 (9)	0.0133 (10)	0.0005 (9)
C5	0.0531 (13)	0.0263 (10)	0.0408 (12)	−0.0050 (9)	0.0123 (10)	−0.0014 (9)
C6	0.0409 (12)	0.0357 (11)	0.0357 (11)	−0.0043 (9)	0.0081 (9)	−0.0010 (9)
C7	0.0417 (12)	0.0291 (10)	0.0361 (11)	0.0041 (9)	0.0131 (9)	0.0011 (9)
C8	0.0424 (13)	0.0396 (13)	0.0738 (17)	0.0033 (10)	0.0125 (12)	−0.0054 (12)
C9	0.0393 (11)	0.0265 (10)	0.0292 (10)	−0.0038 (8)	0.0137 (8)	−0.0023 (8)
C10	0.0339 (10)	0.0323 (10)	0.0264 (9)	−0.0059 (8)	0.0109 (8)	−0.0009 (8)
C11	0.0328 (10)	0.0353 (11)	0.0337 (10)	−0.0048 (8)	0.0121 (8)	−0.0043 (9)
C12	0.0330 (10)	0.0435 (12)	0.0319 (10)	−0.0068 (9)	0.0130 (8)	−0.0106 (9)
C13	0.0374 (11)	0.0503 (13)	0.0278 (10)	−0.0104 (10)	0.0115 (8)	−0.0022 (9)
C14	0.0378 (11)	0.0396 (12)	0.0297 (10)	−0.0058 (9)	0.0096 (8)	0.0041 (9)
C15	0.0378 (11)	0.0350 (11)	0.0318 (10)	−0.0043 (9)	0.0130 (9)	−0.0016 (9)
C16	0.0622 (16)	0.0480 (14)	0.0484 (14)	0.0116 (12)	0.0202 (12)	0.0036 (12)
O14	0.0699 (16)	0.0818 (18)	0.111 (2)	0.0008 (13)	0.0501 (14)	−0.0127 (15)
O15	0.0781 (19)	0.086 (2)	0.186 (3)	−0.0088 (16)	0.006 (2)	0.035 (2)

### Geometric parameters (Å, °)

**Table:**

Cu1—O1	1.9616 (16)	C1—C2	1.509 (3)
Cu1—O7	1.9749 (16)	C2—C3	1.412 (3)
Cu1—O8	1.9637 (15)	C2—C7	1.384 (3)
Cu1—O13	2.0914 (19)	C3—C4	1.403 (3)
Cu1—O2^i^	1.9595 (16)	C3—C8	1.501 (4)
O1—C1	1.253 (3)	C4—C5	1.369 (4)
O2—C1	1.242 (3)	C5—C6	1.378 (3)
O3—N1	1.218 (3)	C6—C7	1.373 (3)
O4—N1	1.207 (3)	C9—C10	1.511 (3)
O5—N2	1.226 (3)	C10—C15	1.383 (3)
O6—N2	1.213 (3)	C10—C11	1.411 (3)
O7—C9	1.243 (2)	C11—C12	1.407 (3)
O8—C9^i^	1.251 (2)	C11—C16	1.499 (3)
O9—N3	1.213 (3)	C12—C13	1.371 (3)
O10—N3	1.226 (3)	C13—C14	1.370 (3)
O11—N4	1.213 (3)	C14—C15	1.380 (3)
O12—N4	1.212 (3)	C5—H5	0.9300
O13—H13B	0.77 (2)	C7—H7	0.9300
O13—H13A	0.80 (3)	C8—H8A	0.9600
O14—H14A	0.81 (3)	C8—H8B	0.9600
O14—H14B	0.80 (3)	C8—H8C	0.9600
O15—H15A	0.83 (4)	C13—H13	0.9300
O15—H15B	0.84 (3)	C15—H15	0.9300
N1—C4	1.482 (3)	C16—H16B	0.9600
N2—C6	1.472 (3)	C16—H16C	0.9600
N3—C12	1.479 (3)	C16—H16A	0.9600
N4—C14	1.465 (3)		
			
O1—Cu1—O7	88.94 (7)	C4—C5—C6	116.6 (2)
O1—Cu1—O8	88.19 (7)	C5—C6—C7	122.0 (2)
O1—Cu1—O13	96.06 (8)	N2—C6—C7	119.5 (2)
O1—Cu1—O2^i^	167.09 (7)	N2—C6—C5	118.6 (2)
O7—Cu1—O8	167.24 (7)	C2—C7—C6	120.1 (2)
O7—Cu1—O13	92.58 (7)	O8^i^—C9—C10	113.81 (17)
O2^i^—Cu1—O7	90.06 (7)	O7—C9—C10	120.09 (17)
O8—Cu1—O13	100.09 (7)	O7—C9—O8^i^	126.09 (19)
O2^i^—Cu1—O8	89.98 (7)	C11—C10—C15	121.60 (18)
O2^i^—Cu1—O13	96.84 (8)	C9—C10—C15	115.34 (17)
Cu1—O1—C1	121.32 (15)	C9—C10—C11	122.91 (17)
Cu1^i^—O2—C1	126.52 (14)	C10—C11—C16	122.00 (18)
Cu1—O7—C9	122.28 (14)	C10—C11—C12	114.66 (18)
Cu1—O8—C9^i^	124.16 (13)	C12—C11—C16	123.33 (19)
Cu1—O13—H13A	123.8 (17)	N3—C12—C13	114.37 (18)
Cu1—O13—H13B	120 (3)	C11—C12—C13	124.97 (19)
H13A—O13—H13B	109 (3)	N3—C12—C11	120.64 (19)
H14A—O14—H14B	124 (4)	C12—C13—C14	117.07 (19)
H15A—O15—H15B	125 (4)	C13—C14—C15	122.0 (2)
O3—N1—O4	124.5 (2)	N4—C14—C13	119.68 (19)
O4—N1—C4	118.1 (2)	N4—C14—C15	118.31 (19)
O3—N1—C4	117.3 (2)	C10—C15—C14	119.52 (18)
O5—N2—C6	118.0 (2)	C6—C5—H5	122.00
O6—N2—C6	118.1 (2)	C4—C5—H5	122.00
O5—N2—O6	123.9 (2)	C2—C7—H7	120.00
O9—N3—O10	124.6 (2)	C6—C7—H7	120.00
O10—N3—C12	117.0 (2)	C3—C8—H8A	109.00
O9—N3—C12	118.34 (19)	H8A—C8—H8B	110.00
O12—N4—C14	118.49 (19)	H8A—C8—H8C	109.00
O11—N4—O12	123.7 (2)	H8B—C8—H8C	109.00
O11—N4—C14	117.8 (2)	C3—C8—H8B	109.00
O1—C1—C2	116.35 (17)	C3—C8—H8C	109.00
O1—C1—O2	125.1 (2)	C12—C13—H13	121.00
O2—C1—C2	118.56 (18)	C14—C13—H13	121.00
C1—C2—C7	116.30 (17)	C10—C15—H15	120.00
C3—C2—C7	120.91 (18)	C14—C15—H15	120.00
C1—C2—C3	122.78 (17)	C11—C16—H16B	109.00
C4—C3—C8	121.0 (2)	C11—C16—H16C	109.00
C2—C3—C8	123.86 (19)	H16A—C16—H16C	109.00
C2—C3—C4	115.0 (2)	H16B—C16—H16C	109.00
N1—C4—C3	119.3 (2)	H16A—C16—H16B	109.00
N1—C4—C5	115.5 (2)	C11—C16—H16A	109.00
C3—C4—C5	125.2 (2)		
			
O7—Cu1—O1—C1	83.36 (17)	O2—C1—C2—C3	9.2 (3)
O8—Cu1—O1—C1	−84.20 (17)	O2—C1—C2—C7	−170.12 (19)
O13—Cu1—O1—C1	175.84 (17)	C1—C2—C3—C4	−175.44 (18)
O1—Cu1—O7—C9	−88.62 (17)	C1—C2—C3—C8	9.3 (3)
O13—Cu1—O7—C9	175.36 (17)	C7—C2—C3—C4	3.8 (3)
O2^i^—Cu1—O7—C9	78.51 (17)	C7—C2—C3—C8	−171.5 (2)
O1—Cu1—O8—C9^i^	87.39 (18)	C1—C2—C7—C6	178.87 (19)
O13—Cu1—O8—C9^i^	−176.78 (17)	C3—C2—C7—C6	−0.4 (3)
O2^i^—Cu1—O8—C9^i^	−79.83 (17)	C2—C3—C4—N1	172.73 (19)
O7—Cu1—O2^i^—C1^i^	−84.61 (18)	C2—C3—C4—C5	−5.4 (3)
O8—Cu1—O2^i^—C1^i^	82.62 (18)	C8—C3—C4—N1	−11.8 (3)
O13—Cu1—O2^i^—C1^i^	−177.22 (17)	C8—C3—C4—C5	170.1 (2)
Cu1—O1—C1—O2	1.7 (3)	N1—C4—C5—C6	−175.0 (2)
Cu1—O1—C1—C2	−176.21 (13)	C3—C4—C5—C6	3.2 (4)
Cu1^i^—O2—C1—O1	−1.3 (3)	C4—C5—C6—N2	−179.9 (2)
Cu1^i^—O2—C1—C2	176.49 (13)	C4—C5—C6—C7	0.8 (3)
Cu1—O7—C9—C10	−177.24 (13)	N2—C6—C7—C2	178.63 (19)
Cu1—O7—C9—O8^i^	2.8 (3)	C5—C6—C7—C2	−2.0 (3)
Cu1—O8—C9^i^—O7^i^	0.1 (3)	O7—C9—C10—C11	48.5 (3)
Cu1—O8—C9^i^—C10^i^	−179.93 (13)	O7—C9—C10—C15	−135.9 (2)
O3—N1—C4—C3	−49.7 (3)	O8^i^—C9—C10—C11	−131.5 (2)
O3—N1—C4—C5	128.6 (3)	O8^i^—C9—C10—C15	44.1 (2)
O4—N1—C4—C3	133.4 (3)	C9—C10—C11—C12	170.77 (18)
O4—N1—C4—C5	−48.3 (3)	C9—C10—C11—C16	−10.4 (3)
O5—N2—C6—C5	8.0 (3)	C15—C10—C11—C12	−4.6 (3)
O5—N2—C6—C7	−172.6 (2)	C15—C10—C11—C16	174.3 (2)
O6—N2—C6—C5	−172.8 (3)	C9—C10—C15—C14	−174.02 (17)
O6—N2—C6—C7	6.6 (3)	C11—C10—C15—C14	1.7 (3)
O9—N3—C12—C11	39.4 (3)	C10—C11—C12—N3	−173.40 (18)
O9—N3—C12—C13	−138.8 (2)	C10—C11—C12—C13	4.5 (3)
O10—N3—C12—C11	−143.2 (2)	C16—C11—C12—N3	7.7 (3)
O10—N3—C12—C13	38.7 (3)	C16—C11—C12—C13	−174.4 (2)
O11—N4—C14—C13	15.9 (4)	N3—C12—C13—C14	176.69 (18)
O11—N4—C14—C15	−162.9 (2)	C11—C12—C13—C14	−1.3 (3)
O12—N4—C14—C13	−165.0 (2)	C12—C13—C14—N4	179.2 (2)
O12—N4—C14—C15	16.2 (3)	C12—C13—C14—C15	−2.1 (3)
O1—C1—C2—C3	−172.83 (19)	N4—C14—C15—C10	−179.30 (19)
O1—C1—C2—C7	7.9 (3)	C13—C14—C15—C10	1.9 (3)

Symmetry codes: (i) −*x*, −*y*, −*z*+2.

### Hydrogen-bond geometry (Å, °)

**Table:**

*D*—H···*A*	*D*—H	H···*A*	*D*···*A*	*D*—H···*A*
O13—H13A···O14^ii^	0.80 (3)	1.84 (3)	2.641 (3)	175 (3)
O13—H13B···O10^iii^	0.77 (2)	2.22 (2)	2.838 (2)	138 (3)
O14—H14A···O15^iv^	0.81 (3)	1.95 (3)	2.758 (4)	172 (3)
O14—H14B···O7	0.80 (3)	2.51 (4)	3.182 (3)	143 (4)
O15—H15A···O12^v^	0.83 (4)	2.16 (4)	2.953 (4)	161 (4)
O15—H15B···O5^vi^	0.84 (3)	2.17 (2)	2.996 (4)	168 (4)
O15—H15B···O6^vi^	0.84 (3)	2.60 (4)	3.273 (4)	138 (4)
C15—H15···O5^vii^	0.93	2.60	3.438 (3)	150

Symmetry codes: (ii) −*x*+1, −*y*, −*z*+2; (iii) *x*, *y*, *z*+1; (iv) *x*−1/2, −*y*+1/2, *z*+1/2; (v) *x*+1, *y*, *z*; (vi) *x*, *y*, *z*−1; (vii) *x*−1/2, −*y*+1/2, *z*−1/2.
